# Detection of circulating tumor DNA in cerebrospinal fluid prior to diagnosis of spinal cord lymphoma by flow cytometric and cytologic analyses

**DOI:** 10.1007/s00277-021-04686-7

**Published:** 2021-10-02

**Authors:** Chisako Iriyama, Kenichiro Murate, Sachiko Iba, Akinao Okamoto, Hideyuki Yamamoto, Ayana Kanbara, Akane Sato, Emiko Iwata, Ryuta Yamada, Masataka Okamoto, Hirohisa Watanabe, Tatsuro Mutoh, Akihiro Tomita

**Affiliations:** 1 Department of Hematology, Fujita Health University School of Medicine, 1-98 Dengakugakubo, Kutsukake-cho, Toyoake, Aichi 470-1192 Japan; 2 Department of Neurology, Fujita Health University School of Medicine, Toyoake, Japan; 3 Department of Analytical Neurobiology, Faculty of Pharmacy, Meijo University, Nagoya, Japan

Dear Editor,

Central nervous system lymphomas (CNSL) are difficult to diagnose. Although flow cytometry (FCM) and cytology using tumor cells in cerebrospinal fluid (CSF) are conventionally performed, the sensitivity is still problematic. Recently, cell-free circulating tumor DNA (ctDNA) has been detected in the CSF of patients with malignancies [1–3]. Here, we report a CNSL showing spinal cord masses in which ctDNA was detectable in CSF with amplicon-based droplet digital PCR (ddPCR) with high sensitivity prior to FCM and cytological diagnosis.

A 62-year-old man presented with a 1-month history of motor/sensory disturbance of the extremities. He had a history of a left orchitis and underwent high orchiectomy 1 year ago. MRI showed enhanced masses in the spinal cord at the C5-7 and Th2-3 level (Fig. 1). A fluoro-deoxy-glucose (FDG)-PET scan showed no additional lesion. His CSF total cell count was 64 × 10^9^/µL, total protein level was 168 mg/dL, and sIL-2R was 251 U/mL. Cytological diagnosis and FCM did not detect lymphoma cells (Fig. 1, CSF-1 and -2). Sequential CSF analysis revealed CD19 + /CD20 + /Ig-lambda + clonal B-cell expansion 1 month later (CSF-3), and a diagnosis of CNSL was made. Systemic and intrathecal chemotherapy and radiotherapy diminished the mass. The B-cell clone in CSF also became undetectable (CSF-4). However, 12 months later, FDG-PET revealed a systemic relapse.

**Fig. 1:**
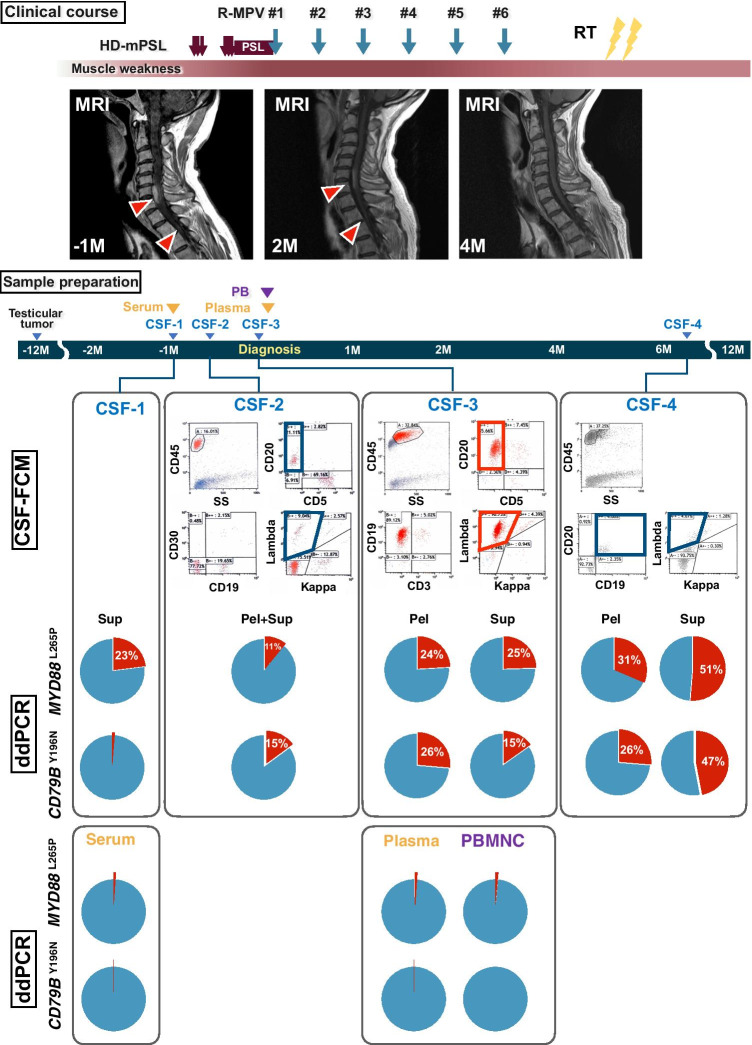
Clinical course of a CNSL patient. Clinical symptoms were observed almost 3 months before diagnosis. Diagnosis of CNSL was made by detecting clonal expansion of Ig-kappa-positive B-cells by FCM using CSF (CSF-3) and cytology. MRI abnormality was detected 1 month before diagnosis (MRI; -1 M). CSF was harvested repeatedly (CSF-1 to CSF-4), and mutational analyses for *MYD88*^L265P^/*CD79B*^Y196^ using an amplicon-based ddPCR strategy were performed using DNA from supernatant (Sup) and pellets (Pel) of CSF. ddPCR using serum/plasma and peripheral blood mononuclear cells was also performed. Benign testicular tumor resection was performed almost 1 year before the CNSL diagnosis. Note that *MYD88*^L265P^/*CD79B*^Y196^ mutations were detected 1 month before diagnosis at the time point of FCM and/or cytology using CSF in which tumor cells could not be detected (CSF-1 and -2). CNSL was confirmed by ddPCR with CSF-cfDNA but not by FCM using CSF-4 during the clinical remission period. Red arrowheads in MRI indicate tumors in the spinal cord. HD-mPSL, high-dose methylprednisolone; PSL, prednisolone; R-MPV, chemo-regimen with rituximab, methotrexate, procarbazine, and vincristine; RT, radiotherapy; CSF, cerebrospinal fluid; M, months

After obtaining an informed consent, we performed *MYD88*^L265P^ and *CD79B*^Y196^ mutational analysis with ddPCR using cell-free DNA (cfDNA) from CSF (Table S1). Gel electrophoresis of DNA from CSF supernatant showed a similar ladder pattern as plasma-cfDNA (Figure S1) [4, 5]. Because of the lower concentration of CSF-cfDNA compared to plasma-cfDNA [6], amplicon-based ddPCR was established (Supplementary methods and Figure S2). *MYD88*^L265P^ and *CD79B*^Y196N^ mutations were detected in both DNA from CSF supernatant and the pellet obtained at diagnosis (CSF-3). Then we performed ddPCR using CSF-cfDNA obtained 24 and 17 days before diagnosis (CSF-1 and -2) and after chemotherapy without obvious clonal B-cell population in FCM (CSF-4). The *MYD88*^L265P^ and *CD79B*^Y196N^ mutations were detected in mostly all the CSF-cfDNA samples analyzed.

We also analyzed DNA from the formalin-fixed paraffin-embedded specimen of the testis obtained 1 year before diagnosis. DNA was extracted from both B-cell rich and sparse lesion. Those mutations were detected only in DNA from the B-cell rich lesion with VAF of 10% and 10.7%, respectively. This phenomenon may suggest that the lymphocytes in his testicular lesion were in pre-lymphoma state.

Our results indicate that tumor DNA in CSF was detected even at the time of negative results with cytology and FCM, and almost 1 month earlier than diagnosis. Genetic analysis with CSF-cfDNA to detect *MYD88/CD79B* mutations may be a more sensitive strategy to detect CNSL than cytology and FCM, even in the period when a pathological diagnosis has not been made. We analyzed the most frequently reported mutations of *MYD88*^L265P^ and *CD79B*^Y196^ [3, 7, 8]. However, about 15% of CNSL do not have these mutations. This necessitates careful interpretation of negative test results. Further careful prospective studies are warranted to determine whether the presence of these mutations in CSF-cfDNA is sufficient evidence to diagnose CNSL.

## Supplementary Information

Below is the link to the electronic supplementary material.

Supplementary file1 (PDF 372 KB)
