# Supplementary material for: Detection of circulating tumor DNA in cerebrospinal fluid prior to diagnosis of spinal cord lymphoma by flow cytometric and cytologic analyses
Source: Ann Hematol. 2021 Oct 2;101(5):1157–9. doi: 10.1007/s00277-021-04686-7 (PMC8993787; doi:10.1007/s00277-021-04686-7)
Supplement: Supplementary file 1 — Supplementary file1 (PDF 372 KB) [file 277_2021_4686_MOESM1_ESM.pdf]

Supplementary information

Supplementary methods

Sample collection

The protocol for the experimental use of patient samples was approved by the institutional review board of Fujita Health University (HG16-032, HG18-017 and HG20-055). CSF was aliquoted into several tubes and centrifuged for 5 minutes at 1,200 ×*g* to obtain supernatant and cell pellets. Total CSF and/or supernatants were directly stored in the freezer at −80°C until further use. Pellets were stored in CELL BANKER 1 (#CB001, TaKaRa. Ohtsu, Japan) and stored at −80°C until further use. Peripheral blood was harvested in collection tubes with EDTA-2Na and stored immediately at 4°C, and plasma and mononuclear cells were harvested.

DNA extraction from CSF, plasma, and peripheral blood mononuclear cells

Cell-free-DNA in CSF and plasma was extracted using the QIAamp Circulating Nucleic Acid Kit (#55114, QIAGEN, Valencia, CA, USA) as described previously [1,2]. The fragmentation condition of cfDNA was confirmed with agarose gel electrophoresis (**Supplementary Figure S1**), and the concentration was measured using a Qubit 4 Fluorometer (Thermo Fisher Scientific, Waltham, MA, USA) as described previously [1,2].

Genetic analysis with ddPCR

Droplet-digital PCR (ddPCR) was performed in accordance with the instruction manual provided by the company (Bio-Rad Laboratories, Hercules, CA, USA). Quantitative detection of mutations was performed using primer probes for *MYD88*^{L265P} (#10049047) and *CD79B*^{Y196} mutations (Y196C, D, F, N, H and S: #10049047).

Plasmid DNAs with wild-type (WT) and mutated *MYD88* toll/interleukin-1 receptor (TIR) domain sequence

The sequence of the WT and L265P-mutated *MYD88* TIR domain was amplified using primer sets MYD88-TIR-2U; 5′-TGG GGT TGA AGA CTG GGC T-3′, and -2L; 5′-GGA CAG GCA GAC AGA TAC-3′, and cloned into the pGEM-T-Easy cloning vector (#A1360, Promega, Madison, WI, USA).

Amplicon-based ddPCR for *MYD88*^{L265P} and *CD79B*^{Y196}

Fragment DNA for the TIR domain of *MYD88* containing the *MYD88*^{L265P} site and the immunoreceptor tyrosine-based activation motif (ITAM) domain containing the *CD79B*^{Y196} site was amplified using PCR primers as follows: MYD88-TIR-4U; 5′-GTT AAC CCT GGG GTT GAA GAC TG-3′, MYD88-TIR-4L; 5′-TAC ATG GAC AGG CAG ACA GAT ACA C-3′, CD79B-ITAM-U; 5′-GAC ACT AAC ACT CTG ATC TCC-3′, and CD79B-ITAM-L; 5′-GAC CAC TTC ACT TCC CCT GT-3′. PCR fragments were purified using the QIAquick PCR purification kit (#28106, QIAGEN), and purified DNA (1×10^{-5} ng/reaction) was used for the ddPCR assay. The mutation-positive cutoff value by the ddPCR using PCR fragments was set at 2%.

References for methods

1. Shimada K, Yoshida K, Suzuki Y, Iriyama C, Inoue Y, Sanada M, Kataoka K, Yuge M, Takagi Y, Kusumoto S, Masaki Y, Ito T, Inagaki Y, Okamoto A, Kuwatsuka Y, Nakatochi M, Shimada S, Miyoshi H, Shiraishi Y, Chiba K, Tanaka H, Miyano S, Shiozawa Y, Nannya Y, Okabe A, Kohno K, Atsuta Y, Ohshima K, Nakamura S, Ogawa S, Tomita A, Kiyoi H (2021) Frequent genetic alterations in immune checkpoint-related genes in intravascular large B-cell lymphoma. Blood 137 (11):1491-1502. doi:10.1182/blood.2020007245
2. Suzuki Y, Tomita A, Nakamura F, Iriyama C, Shirahata-Adachi M, Shimada K, Akashi A, Ishikawa Y, Kaneda N, Kiyoi H (2016) Peripheral blood cell-free DNA is an alternative tumor DNA source reflecting disease status in myelodysplastic syndromes. Cancer Sci 107 (9):1329-1337. doi:10.1111/cas.12994

Supplementary figure legends

Supplementary Figure S1 Fragmented cell-free DNA in plasma and CSF.
cfDNA was harvested from plasma and the supernatant of CSF and separated with 2% agarose gel electrophoresis (lanes 1-3 for plasma obtained from malignant lymphoma patients who are not described in this manuscript, and lane 4 for the supernatant of CSF-3). DNA was harvested from the cell pellet of CSF-3 and also analyzed (lane 5). Note that fragmented DNA was confirmed in cfDNA (red arrows), and larger linear genomic DNA was observed in the cell pellet (green arrow).

Supplementary Figure S2 Optimization of ddPCR conditions using CSF-cfDNA.
(A) The DNA fragment of the TIR domain of *MYD88* cDNA was cloned into a plasmid vector, and the plasmid was utilized as the template DNA for ddPCR of wild-type (WT) *MYD88*. Titration of the DNA concentration was performed to determine the optimal template DNA concentration. **(B)** Plasmids with WT *MYD88* or the mutated $MYD88^{L265P}$ sequence (Mut) were mixed in the indicated ratio with the total concentration of 1×10^{-4} ng/μL and used for the ddPCR assay. **(C)** PCR fragments (amplicons) were obtained using the WT *MYD88* plasmid as template DNA. The concentration of the amplicon was titrated to determine the optimal concentration of template DNA for the ddPCR assay. **(D)** PCR fragments of WT *MYD88* or mutated $MYD88^{L265P}$ were amplified from plasmid DNA with WT and mutated *MYD88* sequences and mixed at the indicated ratio with a total concentration 1×10^{-5} ng/μL. Those amplicons were used in ddPCR to quantitatively determine the VAF.

Supplementary Figure S1

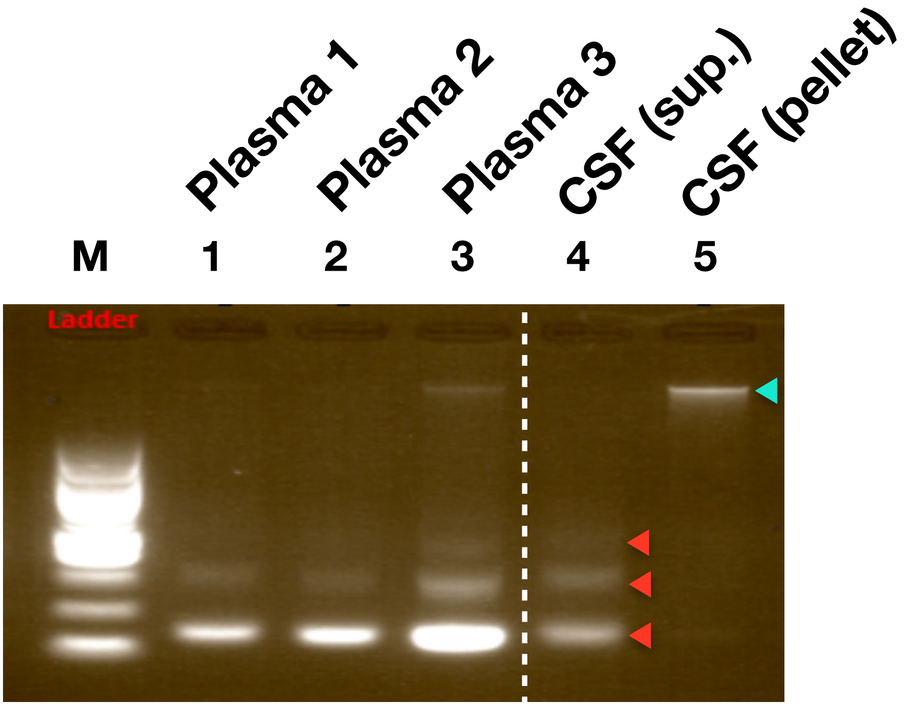

Supplementary Figure S2

A

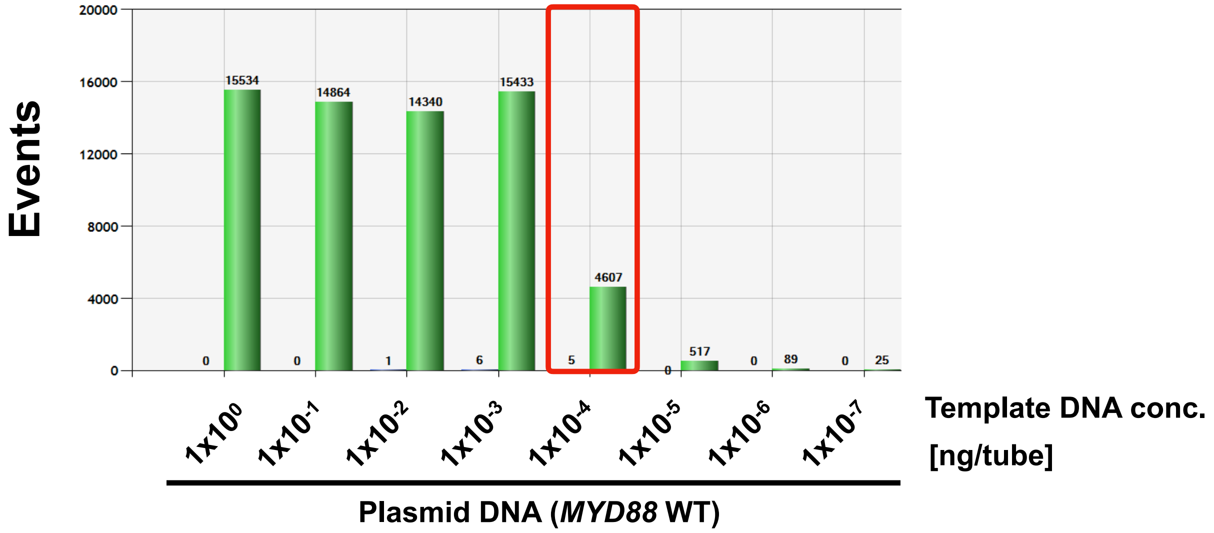

B

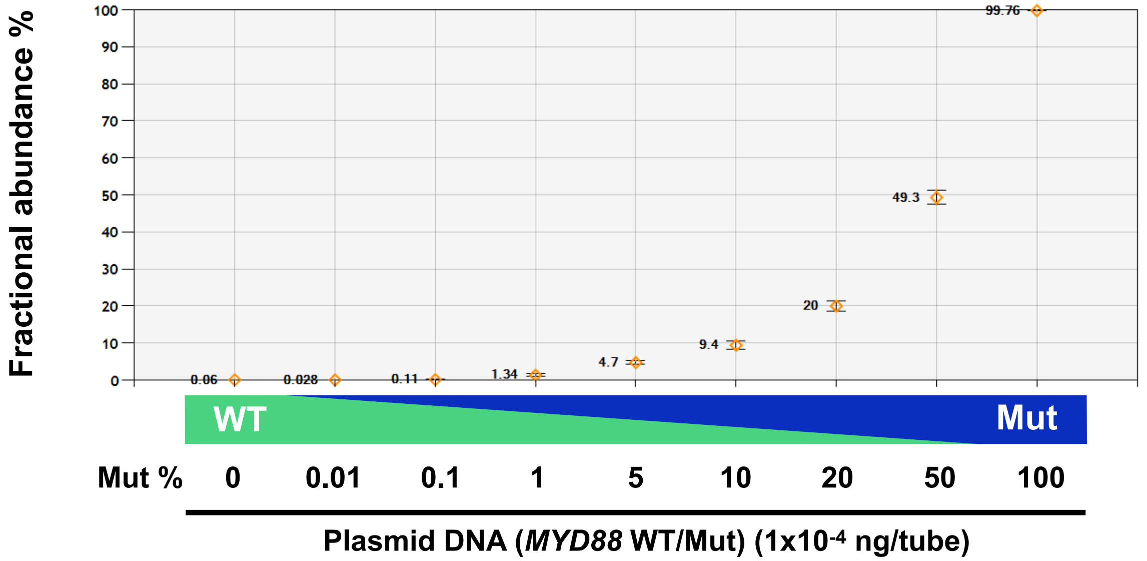

Supplementary Figure S2

C

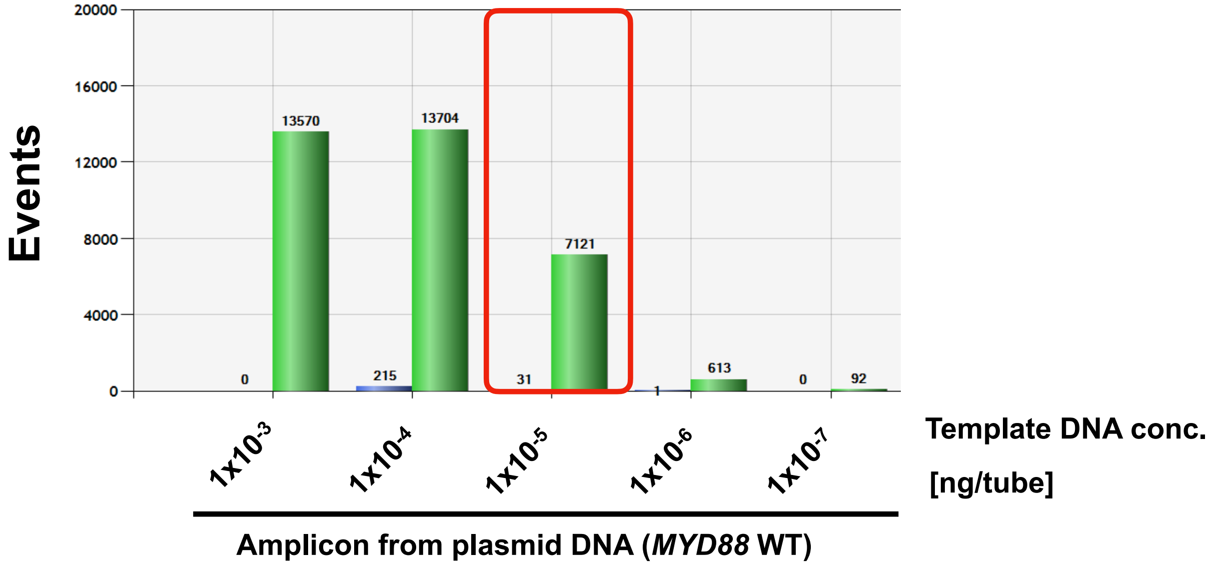

D

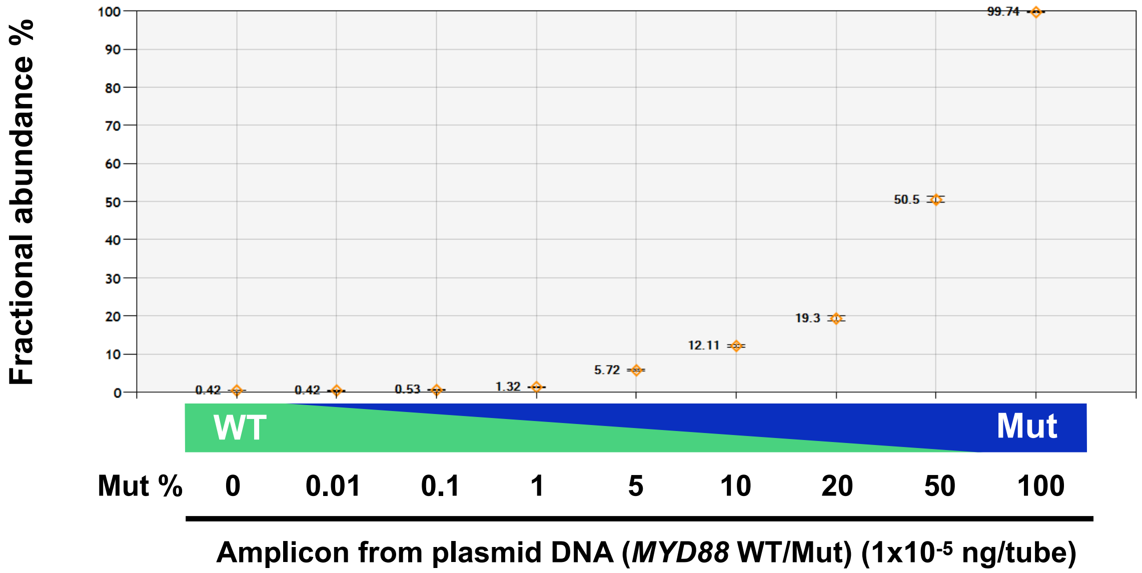

Supplementary Table S1

Detection of tumor cells and DNA using the patient derived materials.

Sample			CSF-1	CSF-2	CSF-3	CSF-4
Duration from diagnosis			-1M	-2W	Diagnosis	6M
Cytology			(-)	(-)	(+/-)	(-)
FCM (clonality)			(-)	(-)	(+)	(-)
CSF-cfDNA concentration [ng/mL]			40.3	Low	28.1	755
Mutation VAF %	CSF-cfDNA	*MYD88* mut	23	11	25	51
		CD79B mut	(-)	15	15	47
	PB-cfDNA	*MYD88* mut	(-)	NA	(-)	NA
		CD79B mut	(-)	NA	(-)	NA
	PBMNC	*MYD88* mut	NA	NA	(-)	NA
		CD79B mut	NA	NA	(-)	NA

M; months, W; weeks, Low; below the lower limit of sensitivity, NA; not available, VAF; valiant allele frequency, mut; mutations.

Negative (+/-) 10-19% 20–29% 40-49% 50-59%
